# Psychosocial Aspects of Delivering Cancer Care to Indigenous People: An Overview

**DOI:** 10.1200/JGO.19.00130

**Published:** 2020-01-13

**Authors:** Gail Garvey, Joan Cunningham, Carole Mayer, Angeline Letendre, Joanne Shaw, Kate Anderson, Brian Kelly

**Affiliations:** ^1^ Menzies School of Health Research, Charles Darwin University, Casuarina, Northern Territory, Australia; ^2^ Health Sciences North Research Institute, Sudbury, Ontario, Canada; ^3^ Cumming School of Medicine, University of Calgary, Calgary, Alberta, Canada; ^4^ Alberta Cancer Prevention Legacy Fund, Alberta Health Services, Edmonton, Alberta, Canada; ^5^ School of Psychology, University of Sydney, Sydney, New South Wales, Australia; ^6^ School of Medicine and Public Health, University of Newcastle, Newcastle, New South Wales, Australia

## Abstract

Globally, a growing body of evidence has reported significant disparities in cancer outcomes between Indigenous and non-Indigenous people. Although some effort is being made to address these disparities, relatively little attention has been directed toward identifying and focusing on the psychosocial aspects of cancer care for Indigenous patients, which are critical components in improving cancer care and outcomes. The purpose of this article is to describe the results of a scoping review of the psychosocial aspects of cancer care for Indigenous people. We highlight considerations in undertaking research in this field with Indigenous people and the implications for clinical practice.

## INTRODUCTION

Cancer disparities in Indigenous populations are of increasing interest globally.^1^ This is reflected in the growing body of research that continues to highlight cancer as a leading cause of illness and death in Indigenous populations. It is well documented that the patterns of cancer care between Indigenous and non-Indigenous patients differ greatly, with Indigenous patients often receiving less optimal treatment.^2-5^ In Australia, Indigenous peoples’ access to and engagement with cancer care is lower at all stages according to clinical guidelines of the cancer continuum, including in screening, timely presentation at diagnosis, continuity of care, compliance with treatment, and survivorship, all of which may contribute to significantly poorer cancer outcomes.^6-8^

**CONTEXT**

**Key Objective**
In light of the significant disparities in cancer outcomes for Indigenous people globally, the aim of this scoping review is to describe the limited evidence base on the psychosocial aspects of cancer care affecting Indigenous people.
**Knowledge Generated**
This paper presents a narrative synthesis of the existing knowledge around the four predominant psychosocial issues reported in the literature that impact Indigenous people with cancer globally: patients’ experiences of care; supportive care needs; quality of life and well-being; and psychological distress.
**Relevance**
This review offers valuable direction for future research and evidence to inform the optimal cancer care and provision of psychosocial cancer care for Indigenous people

Aspects of the broader social environment can also influence (negatively or positively) the way individuals, families, and communities engage with health care and manage their own health.^9^ The social determinants of health (eg, poverty, racism, and lack of a culturally responsive health system) are reported to largely contribute to the cancer burden faced by Indigenous peoples.^10^ Understanding Indigenous peoples’ experience of cancer and engagement with cancer care must consider the cultural contexts and social realities of Indigenous peoples’ lives. These should be reflected in service delivery models and delivery of cancer care. Access to health care services is an important determinant of health outcomes for preventative care and treatment. Indigenous patients living in rural and remote areas have poorer access to cancer treatment and support as a result of the distance from the patients’ homes to the nearest cancer centers. This requires patients with cancer living in these areas to either commute or relocate to an urban center to access treatment.^11,12^ According to the United Nations Report on the State of the Worlds Indigenous Peoples, Indigenous peoples’ access to adequate health care remains a challenging and complex area.^13^

There are known cultural differences in the way many Indigenous peoples understand their health and well-being, perceive cancer, receive and process information about their cancer diagnosis and treatment, and cope with illness.^8,14^ Indigenous peoples define health more broadly than just the physical state of an individual or the absence of disease. In the case of Indigenous Australians, health incorporates the social, emotional, cultural, and physical well-being of an individual’s whole community.^14^ The New Zealand Māori perspective of health includes four cornerstones: te taha wairua (a spiritual dimension), te taha hinengaro (a psychic dimension), te taha tinana (a bodily dimension), and te taha whanau (a family dimension).^15^ In Canada, “the Anishinabek (Ojibway) word mno bmaadis, which translates into living the good life or being alive well, encapsulates beliefs in the importance of balance. All four elements of life, the physical, emotional, mental, and spiritual, are represented in the four directions of the medicine wheel. These four elements are intricately woven together and interact to support a strong and healthy person.”^16(p76)^ These perspectives and concepts of health have important implications for how Indigenous peoples experience and engage with health care. To optimize the benefits of cancer care for Indigenous patients with cancer, care providers need to respond to the psychological, social, and cultural contexts of Indigenous peoples.^17^ However, to date, we know relatively little about the psychosocial impacts on and support needs of Indigenous patients with cancer, which likely affects their engagement with and access to health services.^8^ Research in this area is important to ensure the provision of culturally relevant and appropriate cancer care and reduce the disparities in cancer outcomes for Indigenous peoples.^18^ The purpose of this article is to provide a scoping review of the psychosocial aspects of cancer care and highlight considerations in measuring these for Indigenous peoples.

## METHODS

A scoping review was conducted collaboratively by all authors in PubMed using snowball and citation search methods to identify research describing psychosocial aspects of cancer care for Indigenous peoples.^1^ This approach was taken because of the paucity of research in this field. We explored the published literature on the four predominant topic areas of psychosocial aspects of cancer care for Indigenous patients: patient experience of care, supportive care needs, quality of life (QOL) and well-being, and psychological distress. All manuscripts were published in English, and those with applicable content were obtained and reviewed for their relevance to this scoping review. A narrative synthesis of these four topic areas was conducted and is presented here.^2^

## RESULTS

### Patient-Reported Outcomes and Measures

Increasingly, patient-reported outcomes (PROs) are being used in many countries to assist health professionals in tailoring their health practices and patient care to the individual needs of the patient.^19-21^ PROs include a range of constructs that are reported by the patient, including psychological and physical symptoms, treatment adverse effects (eg, distress, pain, nausea, fatigue), aspects of functioning (eg, role, physical), and multidimensional constructs (eg, health-related QOL [HRQOL]).^20,21^ A number of PRO measures have been developed to assess various aspects of PROs, such as QOL questionnaires,^22^ pain scales,^23^ satisfaction with care surveys,^24^ and unmet supportive care needs tools.^25^

### Experience of Care

Over recent decades, a significant paradigm shift has taken place in the provision of health care toward a patient-centered model of care.^26^ This shift includes interest in measuring patients’ experience of care to determine how well health care is meeting patients’ needs, including their psychosocial needs.^27,28^ Difficulties measuring patients’ experience of care and translating such measures into service delivery improvements are well documented.^27,29,30^ The foundation of patient-centered care, described by the Picker Institute, focuses on understanding and respecting patients’ values, preferences, and expressed needs.^31^ An abundance of tools exist that are underpinned by the patient-centered care principles of access to reliable health care advice, continuity of care, and involvement in decisions.^31^ However, there is great variability in these tools, and their use thus provides limited opportunity to measure patients’ experience of care across institutions and jurisdictions.^27,30^

The extent to which existing tools assess aspects of Indigenous patients’ experience of care that are of value and relevant to Indigenous patients with cancer is poorly understood. Developing measures that are grounded in the views, experiences, and preferences of Indigenous peoples is important to capture their experience of cancer care.^32^ There is growing recognition that different approaches are required to adequately capture and understand the perspectives and experiences of Indigenous patients with cancer.^32^ Australia’s “National Aboriginal and Torres Strait Islander Cancer Framework” recommends the collection and analysis of data about Indigenous patients’ experience of care to ensure the delivery of cancer care that meets the needs of Indigenous peoples.^17^

There is a comprehensive body of qualitative evidence on the experience of care of Indigenous patients with cancer. This evidence describes Indigenous peoples’ cancer experience as a collective journey involving family and friends^33-35^ and an opportunity to draw on strength from their past experiences for emotional and spiritual growth.^34,36^ Patient navigators have provided important emotional and practical support to Indigenous patients.^37,38^ They also have knowledge of the patients’ social and cultural circumstances and the health system, which helps to facilitate trust and engagement in cancer care.^37^ Indigenous peoples’ cancer experience is also fraught with many barriers to receiving comprehensive cancer care. Some barriers include: a lack of access to Indigenous health staff, such as patient navigators; being alienated in the hospital; communication difficulties with health professionals; treatment delays and financial challenges; being away from family and others while receiving treatment; and problems with transportation as well as having to travel long distances.^8,12,33,39,40^

### Supportive Care

Supportive care in cancer settings aims to prevent, reduce, and alleviate the symptoms of treatment; enhance communication between patients and clinicians; and assist patients and their families in managing needs associated with a cancer diagnosis and treatment across a number of interrelated domains.^41^ Typically, these domains include physical needs, psychological needs, social needs, and informational and spiritual needs.^42,43^

An expanding body of evidence demonstrates the value of supportive care approaches in improving experiences and outcomes for those affected by cancer.^42-46^ Although the provision of supportive care across the cancer trajectory is generally of a high standard in some countries (eg, North America and Australia), it is recognized that disparities exist between groups. The National Cancer Control Initiative recommended that “the needs of special populations, especially Aboriginal peoples, be the focus of special efforts to bridge the current gaps in access to and utilisation of culturally sensitive cancer service.”^47(pxvii)^ To assess the supportive care needs of patients, a culturally appropriate needs assessment should be undertaken. Conducting a needs assessment allows a health professional to directly assess the experiences of a patient with cancer, as well as his or her desire for help in specific areas. Needs assessments identify gaps in service provision and can highlight where additional services and resources might be needed.^48,49^ Furthermore, screening for unmet support needs among patients with cancer is considered best practice in some countries and is recommended in optimal cancer care pathways.^45,46,50-53^ Needs assessments have also been associated with enhanced health outcomes, health care cost savings, and better quality service delivery.^41,54,55^ Given the poor cancer prognoses and barriers facing Indigenous patients in accessing cancer treatment and care, it is likely that they may experience specific and high levels of unmet supportive care needs.

Support needs may differ across cultures, and to date, little is known about the specific supportive care needs of Indigenous patients with cancer and their preferences for support throughout their cancer journey. Harris et al^56^ and Burhansstipanov et al^57^ described American Indian and Alaska Native (AI/AN) populations as having substantial unmet educational and resource needs and facing continued inequalities and disparities in accessing culturally and geographically appropriate cancer support, screening, and prevention activities.^57^ In a sample of 248 Indigenous Australian patients with cancer, 71% reported having at least one unmet need. The most commonly reported needs were in the psychological and practical and cultural domains, with money worries being the most frequently reported need.^58^ Harris et al also found substantial unmet needs for financial and human resources to assist AI/AN cancer survivors. In Canada, Gould et al^59^ similarly found that Aboriginal women felt they were denied access to supportive care services because of economic, cultural, language, and literacy disparities between Native and non-Native women.

Routine screening of support needs has the potential to improve cancer care for Indigenous peoples. Gaining a better understanding of the level of unmet needs can assist policy and service development; it also has the potential to reduce disparities in cancer outcomes.

However, in doing so, accurate and culturally relevant needs assessment tools are required, such as the validated Supportive Care Needs Assessment Tool for Indigenous People (SCNAT-IP).^58^ In addition, culturally appropriate training and use of such tools must also be developed; this requires Indigenous community engagement.

### QOL and Well-Being

A cancer diagnosis and subsequent treatment may have a considerable impact on a patient’s QOL.^60^ Increasingly, clinicians have recognized that although traditional end points, such as morbidity and mortality, are important considerations, understanding the impact on patients’ lives more broadly is important in assessing the efficacy of care.^61,62^

HRQOL has been the primary concept used in health care to assess the impact of illness and treatment.^63^ HRQOL is defined as a multidimensional construct that incorporates a person’s perceptions of his or her physical and psychological functioning and social well-being as well as physical symptoms of the disease, treatment, and adverse effects.^62,64,65^ PRO questionnaires have become standard practice for assessing HRQOL.^62,63^

To date, few studies have investigated QOL among Indigenous patients with cancer.^66,67^ In a large study of 596 Native American cancer survivors, Burhansstipanov et al^66^ reported overall QOL was the same for Native and non-Native cancer survivors. Although overall QOL scores were the same for both groups, Native Americans scored lower for physical and social and higher for spiritual QOL domains in comparison with non-Natives. Goodwin et al^68^ reported that American Indian and Native American breast cancer survivors’ social QOL was influenced by the many barriers they faced in accessing treatment and care. In Australia, Indigenous Australians diagnosed with cancer reported a lower overall HRQOL compared with their non-Indigenous counterparts.^69^ Clearly, more research is needed, because QOL data could play an important role in the clinical care of Indigenous cancer survivors and the amelioration of Indigenous cancer disparities. For example, these data could be used to inform patient cancer survivorship care plans or to evaluate the quality of care received by Indigenous cancer survivors.

Although QOL measures have been ubiquitous in clinical settings, it is increasingly recommended that such assessments be broadened to include more general and subjective aspects of well-being.^70^ Given that many Indigenous people regard health holistically and collectively, it is unlikely that existing biomedically focused measures, like QOL, capture Indigenous priorities and worldviews.^71^ Moreover, a review of QOL studies reported few tools included domains specific to Indigenous peoples.^72^ There has been some recent research attention in Australia given to exploring QOL and well-being and the domains valued by Indigenous peoples.^73-77^

The terms QOL and well-being are often used interchangeably, and much ambiguity is evident in the literature around the meanings of these terms.^78^ However, there is an increasing view that the well-being terminology is more cohesive with Indigenous peoples’ understanding of health.^73,78^ In light of the dearth of research in this area, the need to understand well-being and develop associated measures that are relevant to the cultural and social characteristics of Indigenous peoples should be a key priority. Such research will substantially improve the data available to cancer services and policymakers about the psychosocial impacts of cancer and treatment on Indigenous peoples.

### Distress

Not only is cancer a series of different diseases requiring complex treatments, it is also a devastating and traumatic event and a threat to life itself. A significant proportion of patients with cancer at all stages of the disease will experience social and psychological distress and challenges to their emotional well-being as a result of the disease and its treatment.^54^ It is likely that this is also the case for Indigenous patients with cancer, who often present with more complex health and well-being issues.^79,80^

Cancer can affect a person in many ways. The diagnosis may cause fear, anxiety, and depression. Psychological distress, symptoms of cancer, and adverse effects of treatment can have a negative effect on well-being and affect everyday roles and activities.^81^ For example, a cancer diagnosis and subsequent treatment may have an impact on patients’ psychological and physical health, sexuality, body image, finances, relationships, and ability to continue to work and fulfill their role at home.^81-83^ Distress for Indigenous patients with cancer may also originate from a lack of respect and acknowledgment of their cultural beliefs and values or from experiences of racism within the clinical setting.^84,85^ For this reason, it is essential that optimal cancer care incorporates physical and psychological care that considers the cultural and linguistic backgrounds of the patient through the disease trajectory and into survivorship.

Routine screening of patients with cancer for psychological distress using validated measures is supported around the world.^86^ This international support has led to distress being endorsed by the International Psycho-Oncology Society and affiliated organizations as the sixth vital sign in cancer care.^51^ Distress screening has been shown to be acceptable and feasible, and psycho-oncologic interventions have demonstrated small to significant effects on distress.^87,88^ Providing coordinated psychosocial care based on the screening results may benefit patients with cancer experiencing significant distress.^89^ However, to date, we know little about the levels of distress of Indigenous patients with cancer or the cultural applicability of distress screening tools for such patients.

To our knowledge, few studies have explicitly described psychological distress in Indigenous patients with cancer. One Australian study reported one in three Indigenous adult patients with cancer had clinically significant levels of distress (35%; n = 54), but the specific etiologies of distress were unknown.^90^ Another study, conducted in Canada, indicated higher distress levels in Aboriginal/Métis versus non-Aboriginal/non-Métis patients receiving new radical or palliative treatment.^91^ Disparities in cancer care for Indigenous peoples may also include cultural, geographic, and socioeconomic barriers to receiving psychosocial interventions to assist with distress.^84,92^ Given these disparities, it is paramount that we gain a more comprehensive understanding of the psychological distress experienced by Indigenous patients with cancer to ensure their optimal management and care. Developing and evaluating culturally sensitive psychological support and interventions for Indigenous cancer survivors to ensure their optimal management and care are also required.

## DISCUSSION

The importance of considering the various psychosocial impacts on patients with cancer in guiding cancer care is increasingly being recognized and enacted. Although progress in understanding the impacts of care and developing relevant tools for the general population has been rapid, limited attention has been given to understanding the cultural contexts and social circumstances affecting psychosocial aspects of cancer and cancer care for Indigenous peoples. Given the significant disparities in cancer outcomes for Indigenous peoples globally, there is a pressing need to better understand and address psychosocial aspects of cancer care for Indigenous peoples to ensure optimal cancer care in these populations.

Research in this area and the development of measures for Indigenous populations must take into account the social and structural determinants of health affecting many Indigenous peoples. Furthermore, not only must findings, tools, and interventions be relevant to Indigenous peoples, they must also be guided by or developed in significant consultation with Indigenous peoples. This will ensure that research, policy, and practice are determined as important and beneficial to Indigenous peoples by Indigenous peoples.
